# Analysis of COVID-19 Cases' Spatial Dependence in US Counties Reveals Health Inequalities

**DOI:** 10.3389/fpubh.2020.579190

**Published:** 2020-11-12

**Authors:** T. Saffary, Oyelola A. Adegboye, E. Gayawan, F. Elfaki, Md Abdul Kuddus, R. Saffary

**Affiliations:** ^1^Department of Mathematics, Engineering and Computer Science, Chemeketa Community College, Salem, OR, United States; ^2^Evolution Equations Research Group, Ton Duc Thang University, Ho Chi Minh City, Vietnam; ^3^Faculty of Mathematics and Statistics, Ton Duc Thang University, Ho Chi Minh City, Vietnam; ^4^Department of Statistics, Federal University of Technology, Akure, Nigeria; ^5^Department of Mathematics, Physics and Statistics, Qatar University, Doha, Qatar; ^6^Department of Mathematics, University of Rajshahi, Rajshahi, Bangladesh; ^7^Department of Anesthesiology, Perioperative and Pain Medicine, Stanford University, Stanford, CA, United States

**Keywords:** coronavirus, COVID-19, spatial autocorrelation, health rankings, neighborhood

## Abstract

On March 13, 2020, the World Health Organization (WHO) declared the 2019 coronavirus disease (COVID-19) caused by the novel coronavirus SARS-CoV2 a pandemic. Since then the virus has infected over 9.1 million individuals and resulted in over 470,000 deaths worldwide (as of June 24, 2020). Here, we discuss the spatial correlation between county population health rankings and the incidence of COVID-19 cases and COVID-19 related deaths in the United States. We analyzed the spread of the disease based on multiple variables at the county level, using publicly available data on the numbers of confirmed cases and deaths, intensive care unit beds and socio-demographic, and healthcare resources in the U.S. Our results indicate substantial geographical variations in the distribution of COVID-19 cases and deaths across the US counties. There was significant positive global spatial correlation between the percentage of Black Americans and cases of COVID-19 (Moran *I* = 0.174 and 0.264, *p* < 0.0001). A similar result was found for the global spatial correlation between the percentage of Black American and deaths due to COVID-19 at the county level in the U.S. (Moran *I* = 0.264, *p* < 0.0001). There was no significant spatial correlation between the Hispanic population and COVID-19 cases and deaths; however, a higher percentage of non-Hispanic white was significantly negatively spatially correlated with cases (Moran *I* = –0.203, *p* < 0.0001) and deaths (Moran *I* = –0.137, *p* < 0.0001) from the disease. This study showed significant but weak spatial autocorrelation between the number of intensive care unit beds and COVID-19 cases (Moran *I* = 0.08, *p* < 0.0001) and deaths (Moran *I* = 0.15, *p* < 0.0001), respectively. These findings provide more detail into the interplay between the infectious disease and healthcare-related characteristics of the population. Only by understanding these relationships will it be possible to mitigate the rate of spread and severity of the disease.

## Introduction

In December 2019, a cluster of pneumonia cases with unknown etiology were reported in Wuhan, China (1, 2). Following severe acute respiratory syndrome coronavirus (SARS-CoV) originating in China in 2003 and Middle East respiratory syndrome coronavirus (MERS-CoV) originating in Saudi Arabia in 2012, SARS-CoV-2 appeared to become the third and significantly more lethal member of the usually less dangerous family of pathogens (3). COVID-19 caused by SARS-CoV-2 was declared a pandemic on March 11, 2020 (4). Despite previous epidemics, the COVID-19 pandemic has revealed ill-preparedness in many countries (5), especially in the US, with regards to adequate healthcare resources, including hospital beds, ventilators, and personal protective equipment for healthcare workers. The human and economic cost incurred thus far has been devastating.

SARS-CoV-2 belongs to a family of large RNA viruses that are the second most common cause for the common cold in humans. The first cases of COVID-19 were linked to a live animal market in Wuhan, China; however, the current rapid spread is via human-to-human transmission. Our understanding of the pathogenesis still appears to be rudimentary with new data emerging almost daily. COVID-19 primarily spread via respiratory droplets and has an incubation period of up to 14 days, with symptom onset occurring by 11.5 days in ~97.5% of cases (6). Viral shedding occurs mainly from the upper respiratory tract even in asymptomatic patients; thereby, making it difficult to institute preventative measures that rely on symptomatology (7, 8). This has led to different transmission rates from previous outbreaks, and while SARS was essentially under control within 8 months, the trajectory for COVID-19 appears to be significantly different. The disease has a wide variety of presentations ranging from mild cough and fever to shortness of breath, malaise, and even anosmia (9). The severity of the illness is also widespread with the majority of affected individuals being asymptomatic or only showing mild symptoms while others develop severe forms necessitating hospitalizations and even prolonged intubations. Treatment thus far has mainly focused on supportive measures.

The most recent study on COVID-19 risk factors shows a higher probability of infection in elderly individuals as well as men, non-white and individuals from areas with lower socio-economic status or higher population density (10). Initial studies showed COVID-19 to be associated with older age, high population density, kidney disease, obesity, respiratory infections, hypertension, diabetes, cardiovascular diseases and ethnicity (3, 11–14).

To reduce the spread of infections many governments have instituted public health response measures such as movement restrictions and social distancing (15–17). However, while the learning curve regarding the disease process is steep and the country-level (macro) forecasting of the infection spread under different restriction modes is becoming more accurate, more granular analysis at a regional level that takes into account the health rankings of the area has been limited. This is largely due to data availability issues, with data on regional indicators rarely being available in real-time. Furthermore, neighborhood characteristics may also result in higher infection rates and poorer health outcomes in disadvantaged populations and areas because of a lack of public trust in government and health authorities (5) as well as limited access to or use of healthcare (18, 19). During previous epidemics and pandemics, researchers have reported that targeting of commuters from high-incidence locations (20), and low socio-economic areas (21–23) can mitigate disease transmissions in these communities.

In this study, we hypothesized that the number of COVID-19 cases and deaths vary geographically across US counties and that spatial dependencies exist between the disease and demographic, health, behavioral, and clinical care. For example, we also hypothesized that the number of primary care physicians (PCPs) is inversely proportional to the number of COVID-19 cases and deaths while the other variables could be directly proportional. Therefore, we investigated the relationship between socio-demographic and healthcare resources at the county level and the associated infection rate of COVID-19 in the U.S using global and local Moran's Index. In particular, we examined whether the geographic distribution of variables such as race/ethnicity and number of active PCPs, nurses, and hospital intensive care units (ICU) beds had any effect in overall incidence and mortality due to COVID-19.

## Methods

### Data Sources

This study was based on three data sources. The primary source for confirmed cases of and deaths due to COVID-19 in each U.S. County by May 22, 2020 (122 days) were obtained from www.usafacts.org (24). The second source was Kaiser Health News (KHN) (25) for data on the number of ICU beds. Lastly, socio-demographic and healthcare resources were extracted from the County health rankings key findings report 2020 (26, 27). Variables extracted from these data sources were compiled from multiple sources such as the World Health Organization (WHO), U.S. Department of Agriculture, U.S. Census Bureau, governmental, non-governmental, and educational institutions. See Table 1 for the list of variables and their sources.

**Table 1:** Descriptive summary of dependent and independent variables considered in this study.

**Variable**	**Description**	**Source**
**Outcome variables**		
Counts of COVID-19 cases/deaths	Number of confirmed cases/deaths of COVID-19 in each county per 100,000 population	(24)
**Health, behaviors and clinical care**		
ICU beds	Number of ICU beds	(25)
PCP	PCPs per 10,000 population	(26)
Adult obesity	Percentage of adults with Body mass index (BMI) > 30	(26)
Diabetes	Percentage of adults aged 20 and above with diagnosed diabetes	(26)
Uninsured	Percentage of people under age 65 without insurance	(26)
Flu vaccinations	Percentage of annual Medicare enrollees having an annual flu vaccination, overall and subgroups	(26)
**Demographics**		
Black American	Percentage of the population that is non-Hispanic Black or African American	(26)
Hispanic	Percentage of the population that is Hispanic	(26)
White	Percentage of the population that is non-Hispanic white	(26)

The boundary file of the 3,142 U.S. Counties was obtained from the U.S. Census Bureau, 2018 cartographic boundary files (www.census.gov). Due to the spatial nature of our study, we included only 3,108 contiguous counties after excluding counties in Alaska and Puerto Rico.

We investigated the potential effects of county-level factors including the number of hospital beds, ICU beds, number of PCPs, adult obesity, number of uninsured, number of flu vaccinations, and race on the incidence of and deaths from COVID-19. The dataset used in this study can be downloaded from https://github.com/oyeadegboye/USA_COVID-19.

### Data Analysis

Descriptive summaries of study characteristics were presented as mean, median and interquartile range. Often in spatial data, there is some degree of dependency among observations within a geographical space (28, 29); therefore, we measured the bivariate spatial autocorrelation between county-level health factors and the number of confirmed COVID-19 cases using Moran's Index based on Queen's contiguity spatial-lag of order 1 (immediate neighbors) (30). Moran's I is the most common measure of global spatial autocorrelation which gives the overall distribution of departures from randomness. We presented both global and local Moran's *I* (univariate and bivariate) and local indicators of spatial association (LISA) (31) at the county level to provide information on spatial clusters and outliers and types of spatial correlation. LISA allows for the decomposition of global Moran's I inferring the scope of clustering in smaller areas (31). Similarly, LISA calculation was based on Queen's contiguity spatial-lag of order 1, and the statistical significance of the pattern of spatial autocorrelation in each county (relative to the entire spatial scope) was tested at 5% level of significance. The counties' spatial dependence was plotted on the map and color-coded according to the type of interaction. The high-high and low-low areas represent positive local spatial correlations that are identified as spatial clusters (red and blue color, respectively), while the high-low and low-high areas represent negative local spatial correlations that are classified as spatial outliers (pink and light blue color, respectively).

All statistical analyses were implemented in R statistical software version 3.6.2 (32).

## Results

**Figure 1:**
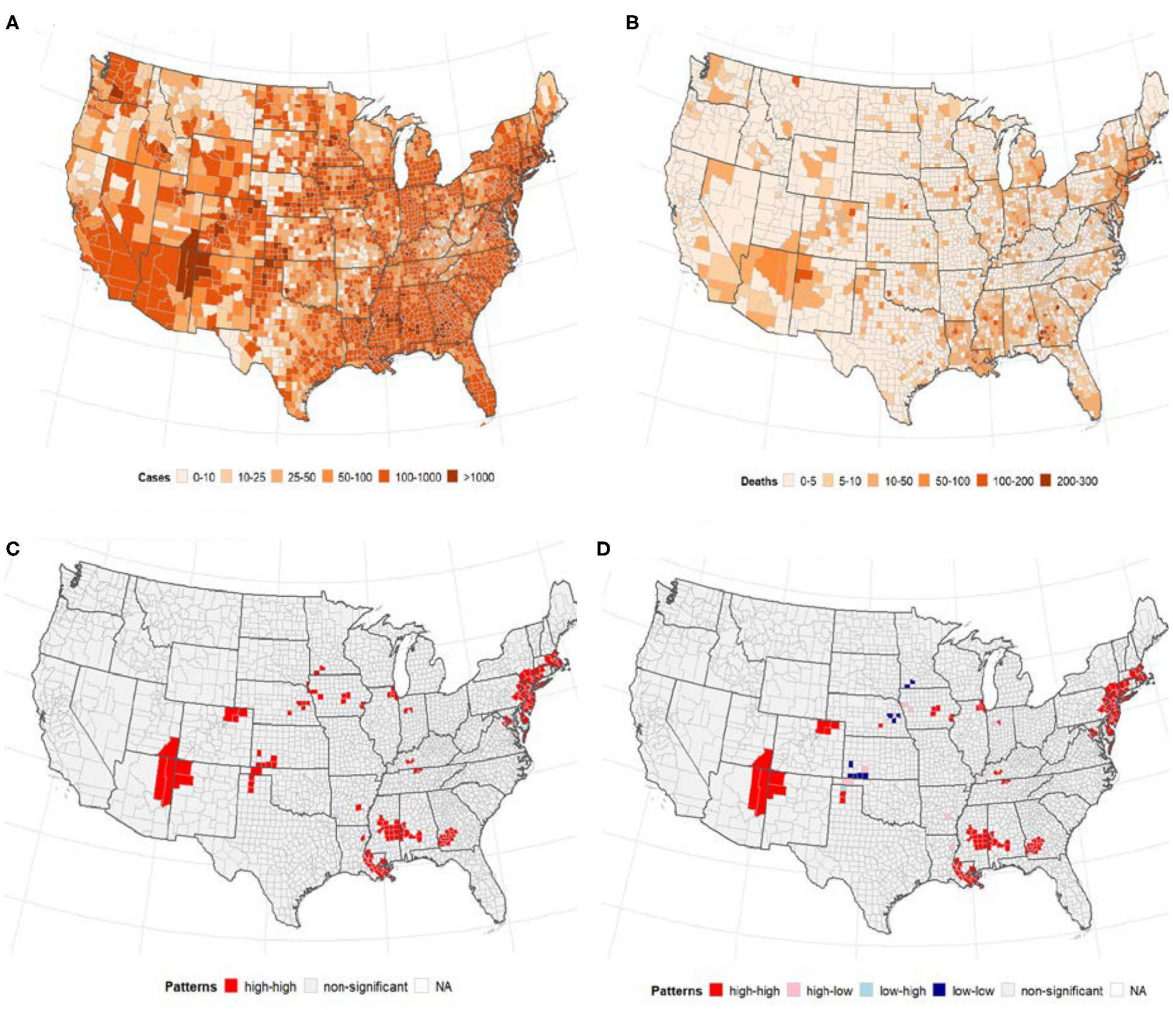
The number of confirmed cases of COVID-19 and deaths per 100, 000 population by US county. Note: Visual effects may be distorted due to the large area of US counties in the West compared to the East. **(A)** Cases/100,000 population. **(B)** Deaths/100,000 population. **(C)** Univariate LISA cases/100,000. **(D)** Univariate LISA deaths/100,000.

A total of 1,622,612 cases of confirmed COVID-19 and 97,087 deaths were recorded in the US as of May 24, 2020. Figure 1 shows the distribution of the number of confirmed cases of COVID-19 and deaths in the 3,108 contiguous counties considered in this study. The number of cases per 100,000 revealed a clear separation between the West and East due to much few cases in the central counties (Figure 1A). A similar pattern was observed in the deaths per 100,000, with higher rates observed in the East (Northeast and Southeast). Table 2 presents the exploratory summaries of incidence and deaths from COVID-19 and county level health factors. An average of 503.1 cases and 30.5 deaths due to COVID-19 per county were recorded during the study period. We observed moderate and high significant global spatial autocorrelation based on Queen Contiguity spatial-lag of order 1 in the distribution of COVID-19 cases and deaths across US counties (Global univariate Moran's *I* =0.228, *p* < 0.0001), and (Global univariate Moran's *I* =0.477, *p* < 0.0001), respectively. Univariate LISA plots displayed the presence of significant spatial clusters or outliers by county (Figures 1C,D). Counties with similar numbers of cases/deaths are clustered with immediate neighboring counties, especially in the Northeast and Southeast.

**Table 2:** Descriptive summary of dependent and independent variables considered in this study.

**Variables**	**Mean**	**Median (IQR)^a^**	**Global bivariate Moran's I**	
			**Deaths**	**Cases**
Cases per 100,000 population	503.1	32.0 (7.0–147.8)		0.228^****^^b^
Deaths per 100,000 population	30.5	1.00 (0–5)	0.476^****^^b^	
**Health, behaviors, and clinical care**				
ICU beds	23.6	0 (0–12.0)	0.15^****^	0.08^****^
PCPs	54.5	48.0 (32.0–71.0)	0.001	−0.01
Adult obesity	32.9	33 (29–37)	0.005	0.01
Diabetes	12.2	12 (9–15)	0.03	0.01
Uninsured	11.5	11 (7–14)	0.005	0.012
Flu vaccinations	41.7	43 (36–49)	−0.004	−0.006
**Race/ethnicity**				
Black American	9	2.2 (0.7–10.2)	0.264^****^	0.174^****^
Hispanic	9.6	4.4 (2.4–10.0)	−0.002	0.008
Non-Hispanic white	76	83.4 (64.3–92.3)	−0.137^****^	−0.203^****^

a *median and interquartile range over 3,142 US Counties*.

b *univariate Moran's I*.

Significant at ^*^p < 0.05, ^**^p < 0.01, ^***^p < 0.001,

**** *P < 0.0001*.

### ICU Beds and Number of PCPs

**Figure 2:**
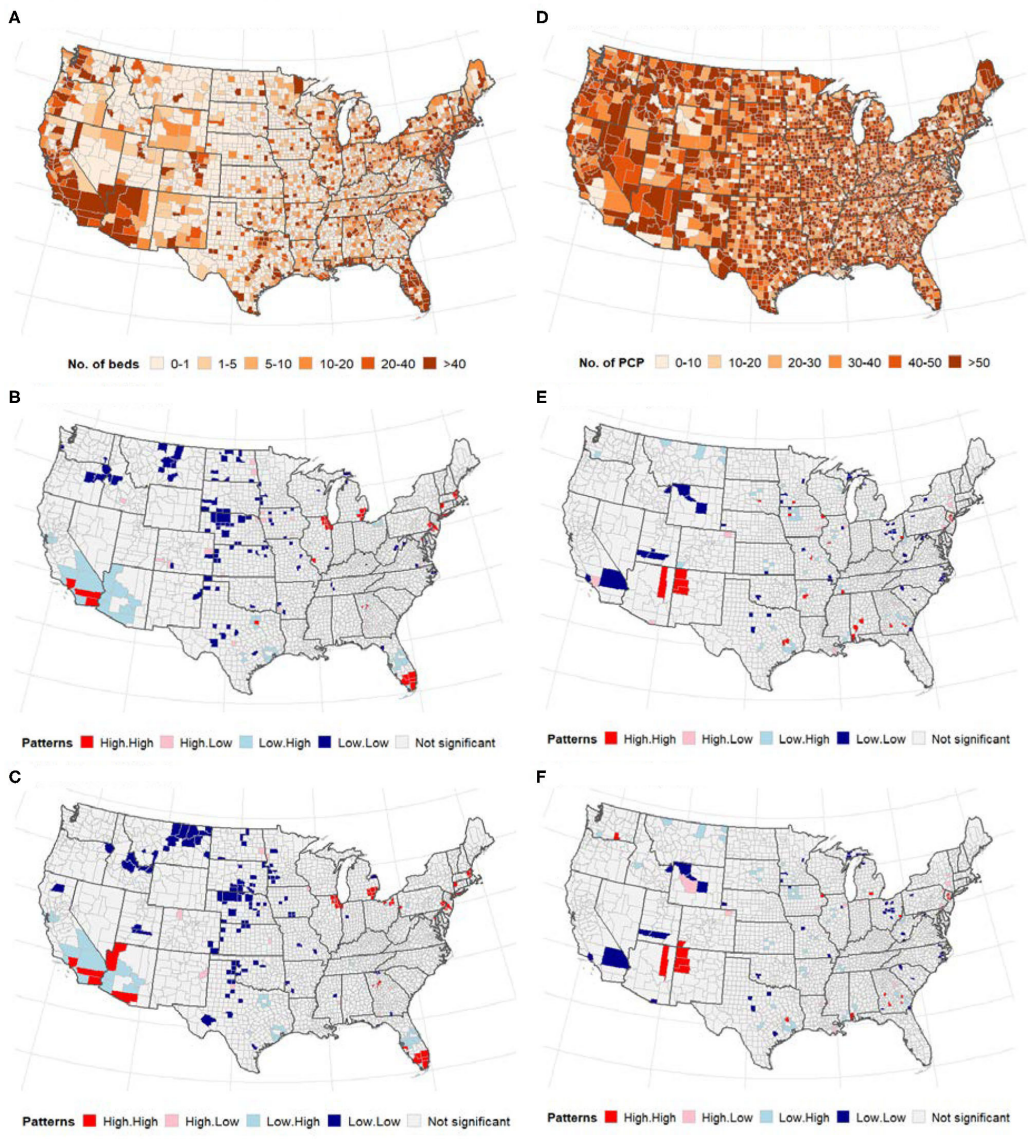
(Top) Distribution of the number of ICU beds **(A)** and PCPs **(D)** across US counties; (middle and bottom) Bivariate LISA map between ICU beds/PCPs and COVID-19. **(A–C)** ICU beds, **(D–F)** PCPs. The high-high and low-low areas represent spatial clusters, while high-low, and low-high represent discordant patterns. **(A)** Number of intensive care units (ICU) beds. **(B)** Cases vs. ICU beds. **(C)** Deaths vs. ICU beds. **(D)** Primary care physicians (PCP)/10,000 population. **(E)** Cases vs. physicians. **(F)** Deaths vs. physicians.

Global bivariate Moran's *I* identified a significant but weak spatial dependence between COVID-19 cases and the number of ICU beds (Moran's *I* = 0.08, *p* < 0.0001). Similarly, we observed weak global bivariate spatial dependence between the number of COVID-19 deaths and the number of ICU beds (Moran's *I* =0.15, *p* < 0.0001). However, there was no significant global bivariate spatial dependence between the disease and the number of PCPs (Moran's *I* =0.0001, *p* > 0.05) and COVID-19 deaths and PCPS (Moran's *I* = −0.01, *p* > 0.05). Figure 2 displays the maps showing the number of ICU beds (Figure 2A), the number of PCPs per county (Figure 2D) and the spatial correlation analysis (middle and bottom panels of Figure 2). We observed a high concentration of ICU beds in the Northeast, South Atlantic and West Pacific (Figure 2A). Local bivariate Moran's *I* analyses are displayed as LISA maps (Figures 2B,C). Few spatial clusters of low COVID-19 cases (and deaths) with a low number of ICU beds were observed in the Midwest counties with few high-high relationships noted in the Northeast, South Atlantic and West Pacific (Figures 2B,C). There was no clear spatial pattern for the number of PCPs (Figure 2D) although a few clusters of high-high and low-low were observed in the Midwest.

### Adult Obesity and Diabetes

**Figure 3:**
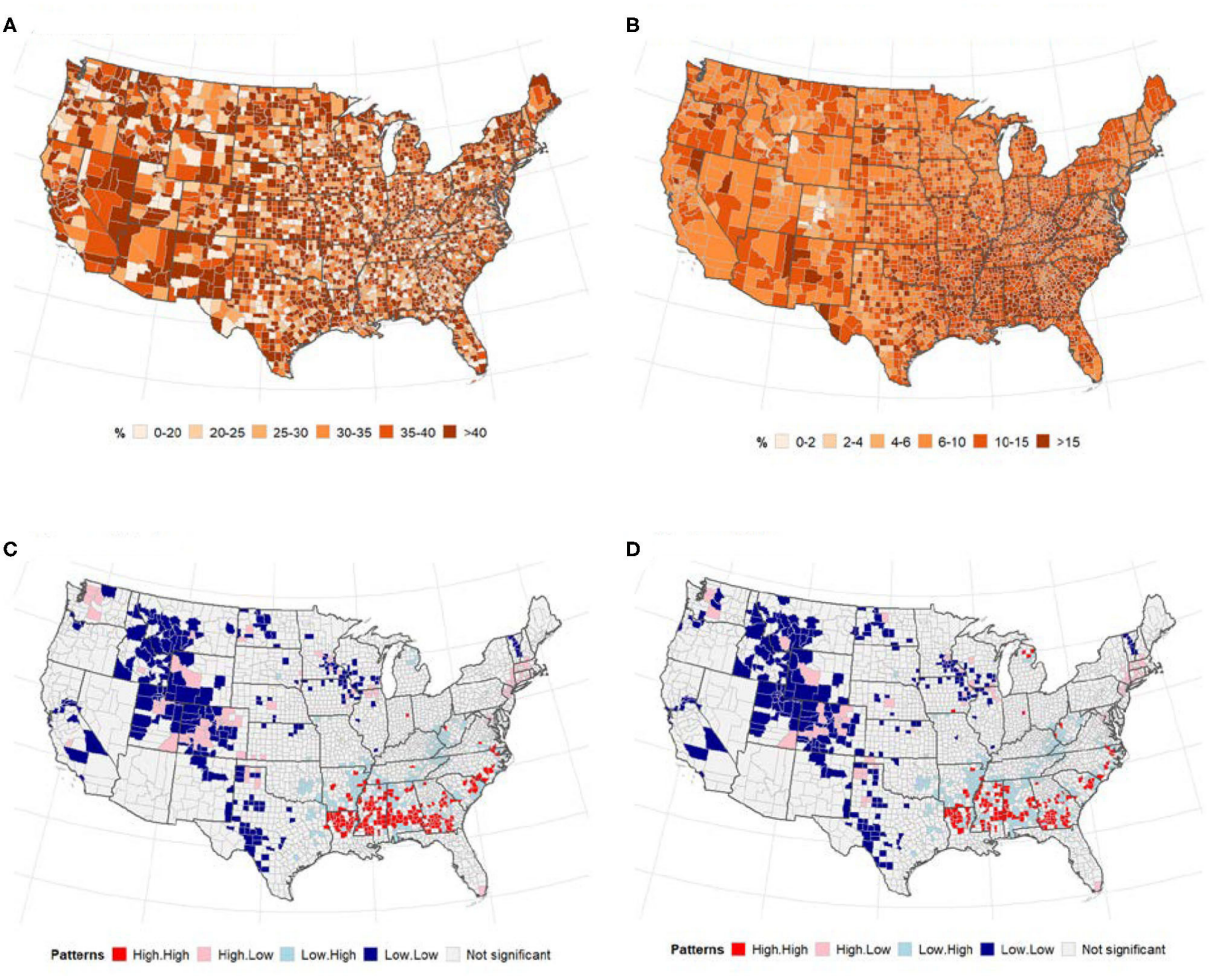
Relationships between adult obesity (right), diabetes (left) and number of COVID-19 cases (and deaths). Spatial distribution of **(A)** adult obesity and **(B)**, diabetes. LISA map for spatial dependence between adult diabetes and COVID-19 cases/deaths **(B,C)**. **(A)** % of adults with BMI > 30. **(B)** % of adults aged 20 and above with diagnosed diabetes. **(C)** Cases vs. diabetes. **(D)** Deaths vs. diabetes.

Figures 3A,B presents the spatial distribution of adult obesity measured by BMI > 30 and diabetes across US counties. There was a significant spatial dependency in the incidence of adult obesity (Global univariate Moran's *I* = 0.01, *p* < 0.3341) and diabetes (Global univariate Moran's *I* = 0.34, *p* < 0.0001). However, there was no significant global bivariate spatial correlation in the number of cases (and deaths) of COVID-19 vs. adult obesity (*p* > 0.05). Similarly, although the global spatial correlation between diabetes and COVID-19 cases/death was not significant, several counties in the Midwest and South of the U.S. were identified as significant low-low and high-high clusters for COVID-19 cases/deaths and diabetes (Figures 3C,D).

### Flu Vaccination and Health Insurance

**Figure 4:**
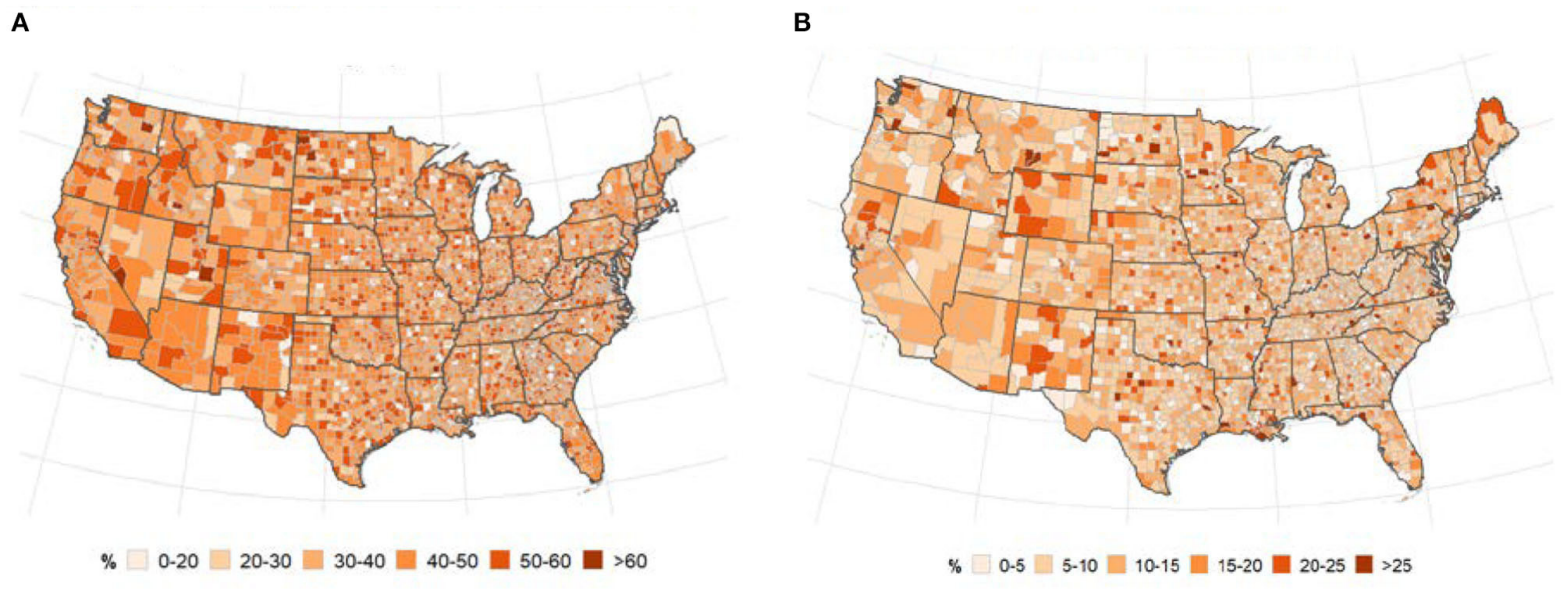
Spatial distribution of flu vaccination **(A)** and uninsured population **(B)**. **(A)** % of annual medicare enrollees having an annual flu vaccination, overall and subgroups. **(B)** % of people under age 65 without insurance.

The geographical distribution of uptake of flu vaccination and uninsured population is displayed in Figures 4A,B. There was a slightly higher increase in flu vaccination uptake in Eastern Pacific counties while the percentage of uninsured population was slightly higher in the Midwest. Global bivariate Moran's *I* indicated no spatial autocorrelation between COVID-19 and flu vaccination or percentage of uninsured population (Table 2).

### Race

**Figure 5:**
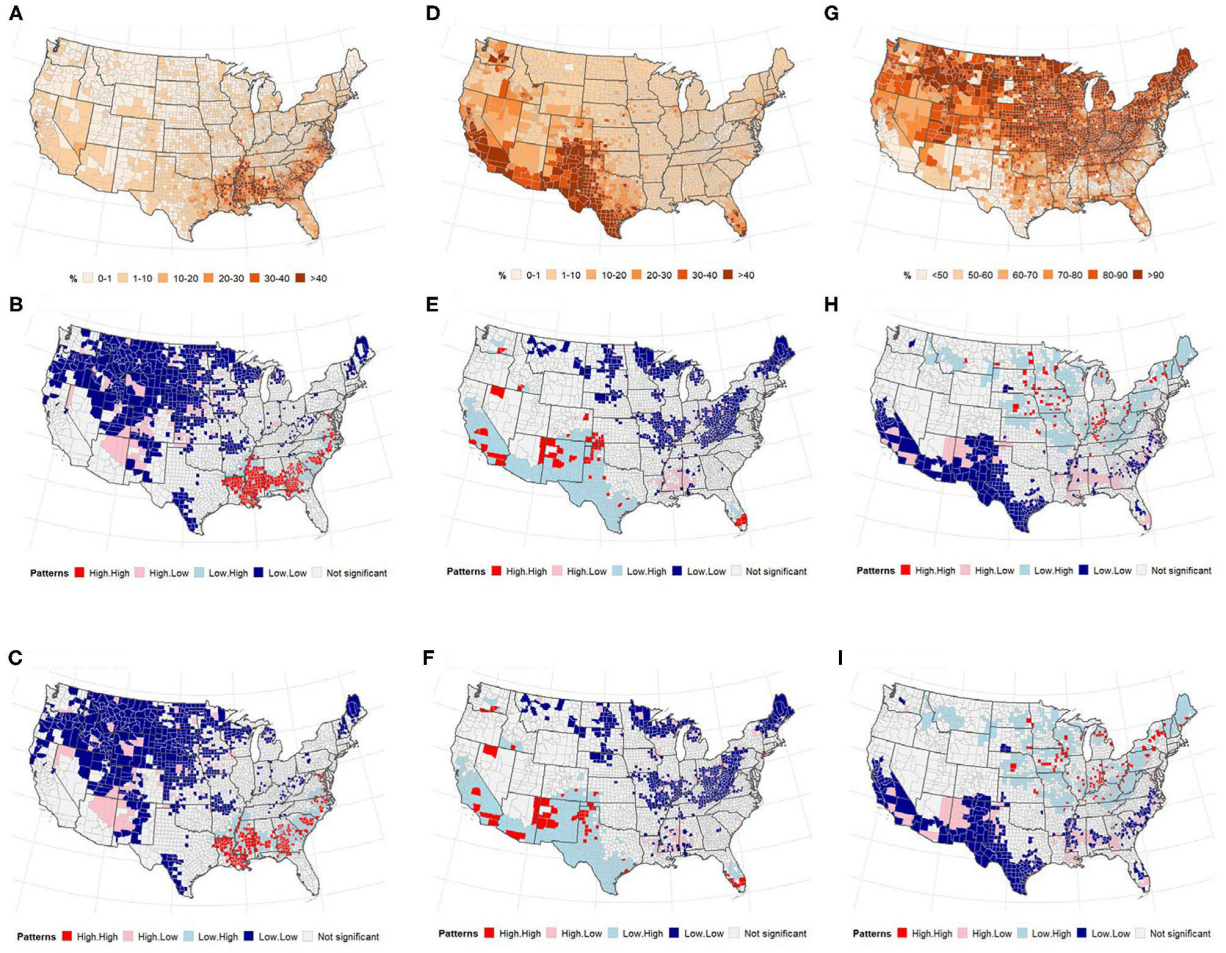
(Top) Percentage of population, (middle and bottom) LISA map for spatial dependence between race, and COVID-19 incidence. **(A–C)** non-Hispanic Black or African American **(D–F)**, Hispanic. **(A)** % non-Hispanic Black or african american. **(B)** Cases vs. Blacks. **(C)** Deaths vs. Blacks. **(D)** % of population that is Hispanic. **(E)** Cases vs. Hispanic. **(F)** Deaths vs. Hispanic. **(G)** % of population that is non-Hispanic White. **(H)** Cases vs. White. **(I)** Deaths vs. White.

Figure 5 presents the distribution of the US population by race and local patterns of spatial correction at the county level between COVID-19 cases and deaths. The global bivariate Moran's *I* for the spatial correlation between the percentage of Black American population and COVID-19 cases and deaths were 0.174 (*p* ≤ 0.0001) and 0.264 (*p* ≤ 0.0001), respectively (Table 2). Global bivariate Moran's *I* identified significant spatial dependence clusters between COVID-19 cases/deaths and Black American and non-Hispanic white populations across U.S. counties. The global spatial autocorrelation between COVID-19 cases (and deaths) was negative for the non-Hispanic white population and positive for the Black American population (Table 2). The LISA map (Figure 5, right panel) displayed the clusters of these relationships. The high Black American population and high COVID-19 cases/deaths as well as low Black American population and low COVID-19 cases/deaths are significant in the Southeast. The West and Midwest revealed many outliers of high cases/deaths but a low Black American population. The global spatial correlation between the distribution of Hispanic population and COVID cases/deaths were not significant (*p* > 0.05); however, there were some significant local clusters of high cases/deaths and high Hispanic population and low cases/deaths and low Hispanic population in the Southwestern and Western U.S. counties. Most of the significant outliers were located in the Mideast, Southeast and Northeast. There were significant clusters of low COVID-19 cases/deaths and low non-Hispanic white populations in the West (South and Pacific) (Figures 5H,I). Significant outlying counties displayed high-low or low-high bivariate correlations between race/ethnicity and COVID-19, and they are illustrated and color-coded as pink and light blue, respectively, in Figure 5.

## Discussion

Containing and ultimately eradicating infectious diseases require interdisciplinary collaboration because the spread not only depends on the nature of the pathogen but also on health and healthcare-related factors as well as socioeconomic characteristics of the population. A better understanding of all these factors and their interdependencies are essential to finding the best strategies to contain the pathogen and protect the population. The purpose of this study is to shed more light on the spread of the COVID-19 pandemic by analyzing county-level relationships between confirmed U.S. COVID-19 infections and deaths and healthcare and socio-demographics indicators such as ICU beds, number of PCPs, prevalence of adult obesity and diabetes, number of uninsured individuals, and flu vaccinations as well as race/ethnicity.

Our study showed a significant positive spatial dependency between confirmed COVID-19 cases/deaths and the number of ICU beds, with pockets of positive autocorrelation predominantly located in Central and Northwestern regions (low-low) and along the Southwestern, Southern, and Northeastern borders (high-high). According to data presented by the Society of Critical Care Medicine, the U.S. have the highest number of ICU beds per 100,000 with significant variations across the country and an estimated 94% of ICU beds in metropolitan areas with populations of >50,000 (33). ICU bed availability also indicates that the hospital is well-equipped to serve as a tertiary care center, treating referred, or transferred patients who require an acute level of care not available at their local hospital. Our data indicate that areas with a high population density have higher transmission rates than areas with lower population density. More rural areas with fewer ICU beds may therefore benefit from the lower population density and a resulting lower transmission rate. Besides, areas with more ICU beds may serve as referral centers for patients with more severe forms of COVID-19 therefore resulting in a higher death rate.

Interestingly, we did not find any significant spatial autocorrelation between the COVID-19 cases/deaths and the number of PCPs, prevalence of adult obesity and diabetes, number of uninsured individuals or flu vaccination. Our initial hypothesis was that the number of PCPs would be inversely proportionate to the number of COVID-19 cases/deaths, while the other variables listed would be directly proportionate. However, the data do not show any significant correlation. Regarding the number of PCPs, this may indicate that the diagnosis of COVID-19 is not commonly made by the PCP. Unlike other chronic diseases, COVID-19 presents with acute respiratory symptoms that may require immediate attention found in the emergency department—particularly if symptoms are severe. In addition, COVID-19 requires a nasal swab for definitive diagnosis. Unfortunately, testing was not widely accessible during the beginning of the pandemic and most PCPs likely had limited access.

COVID-19 has been shown to affect individuals with comorbidities more severely. Among other chronic illnesses, the Centers for Disease Control and Prevention (CDC) listed diabetes and severe obesity (BMI>40) as risk factors for COVID-19 (34). We analyzed data on obesity (BMI>30) and diabetes. Interestingly, the data presented here did not reveal any significant global spatial autocorrelation for these variables; however, the data identified counties in the Midwest and South that were significant low-low and high-high clusters, respectively, for COVID-19 cases/deaths and diabetes. One explanation for the lack of spatial dependency between obesity and COVID-19 may be the fact that we looked at a BMI of >30 rather than >40 (34). It is well-documented that an increase in BMI predisposes patients to more severe chronic illnesses, and although obesity in itself may place patients at a higher risk, it may require severe obesity to see a significant difference in the COVID-19 disease process compared to individuals with a normal BMI. Known as the “stroke belt,” these areas in the southeastern part of the U.S. have been shown to have a consistently higher stroke rate than the rest of the country (35). While the etiology is unknown and likely multifactorial, the finding was still useful in creating targeted public health initiatives to address the issue. A similar approach can be used for COVID-19. Although our data did not show a significant association, between COVID-19 and diabetes and BMI, spatial clusters can be used to identify areas that would benefit from targeted interventions and public health initiatives.

Recent current events have put racial inequality back into the forefront of the national and even international conversation. Unfortunately, it is often overlooked that racial inequality affects all aspects of daily living, including healthcare (36). Limited access to healthcare does not only affect acute conditions, but can also result in chronic disease that worsens over time if left untreated. In the case of COVID-19, this is particularly concerning as inequalities may lead to significant comorbidities, which predispose individuals to the disease for which they may not be able to seek treatment. For example, data from New York City revealed a disproportionately higher death rate due to COVID-19 in Black American and Hispanic persons than white and Asian persons (37). Studies on patients treated at five NHS Hospitals and at Oxford Royal College, UK, showed similar results (38, 39). Our data confirm these findings and showed a positive significant spatial dependence between Black Americans and COVID-19 cases/deaths but negative spatial dependency for non-Hispanic whites. Northwest and Southeast counties with a high proportion of Black Americans are also positively spatial correlated with COVID-19 cases/deaths. The counties with a negative spatial dependency between cases/deaths and non-Hispanic whites are located along the Northern and Southern borders, respectively.

Taken together, these data indicated that the global pandemic affects different races in the U.S. differently, with racial minorities having higher rates of infection and death. There is a multitude of explanations that may account for these findings. In addition to comorbidities due to poor health maintenance and limited access to acute care, living conditions may also lead to a higher risk of COVID-19 infection and death. Racial minorities are more likely to live in more densely populated areas within multi-generational households, making preventative measures, such as social distancing, more challenging or even impossible (37). As previously reported by the WHO, crowding, which occurs when the number of occupants exceeds the capacity of the dwelling space available, has been associated with significant adverse health outcomes, including infectious diseases (40). Unfortunately, mitigating this issue requires local and national governments to provide more adequate housing options that are available to low-income families. In the case of COVID-19, this may also require adequate testing, contact tracing and the ability to assist with isolating individuals who have tested positive and who would otherwise not be able to due to economic restrictions. Additionally, the provision of hand sanitizers and masks to decrease the risk of spread should be considered if isolation is not possible.

This study has several limitations. First, detailed testing data including the number of negatives and positive cases were not available. Particularly during the early phase of the first wave, testing was very limited and unequally distributed throughout the U.S. Therefore, areas that appear to have low COVID-19 cases/deaths may in fact have had higher rates that went unnoticed due to lack of testing. As testing improves, data points may start to represent actual case load more accurately. Secondly, our data did not show any significant correlation between obesity and the rate of COVID-19 cases/deaths. As mentioned above, severe obesity has been associated with an increased predisposition for COVID-19 (8, 34); however, in this data set the cutoff was set at BMI > 30. This may indicate that obesity in itself does not result in higher positive rates but that extremes of obesity have to be achieved to see significant differences. Thirdly, our data did not consider the timing of preventative measures such as shelter-in-place and mandatory mask policies. There were significant differences in when and how different states approached the pandemic. For example, California was among the first states to declare a strict shelter-in-place policy, while other states decided to completely forgo this measure. Likewise, the adaptation of mask policies was very variable throughout the U.S. and may have affected the COVID-19 cases/deaths significantly. Lastly, although, we observed several interesting patterns of spatial dependency in this study, these patterns should be interpreted with caution, because inferences were based on uncorrelated level of significance (0.05). However, we presented supplementary results (Supplementary Table 1) based on correlated tests (multiple comparisons) using Bonferroni bounds (conservative approach) and false discovery rates (less conservative approach) (41–43). Although, there were slight changes to the pattern of the spatial dependency in the LISA map using false discovery rate adjusted alpha level, that of Bonferroni correction were more pronounced. Additional analyses based on recent data would make it possible to reinforce the patterns detected during the early phase of the epidemics.

## Conclusion

By shedding more light on the interplay between COVID-19 and health-related characteristics of the US population, this study can help mitigate the spread rate and severity of the disease as well as advocate for more attention to minorities at higher risk of severe infection and death. Our analysis has shown significant positive spatial dependency between confirmed COVID-19 cases/deaths and the number of ICU beds. Moreover, significant low-low and high-high clusters for COVID-19 cases/deaths and diabetes are shown in the Midwest and South, areas which are known as the “stroke belt” with a significantly higher stroke rate that the rest of the nation. Interestingly, we did not find any significant spatial correlation between the COVID-19 cases/deaths and the number of PCPs, prevalence of adult obesity and diabetes, number of uninsured individuals, or flu vaccination. Last but not least, our data confirm previous findings that racial minorities are at higher risk of severe infection and death, in particular, a positive significant spatial dependence between Black Americans, and COVID-19 cases/deaths is shown which could be explained with poor living standards, insufficient access to healthcare facilities, and domicile in areas with higher population density.

## Data Availability Statement

The datasets used in this study can be downloaded from https://github.com/oyeadegboye/USA_COVID-19.

## Author Contributions

TS and OAA: conceptualization and investigation. TS, OAA, and MK: data curation. OAA: formal analysis and methodology. RS: project administration and resources. OAA, EG, and FE: visualization. TS, OAA, MK, EG, FE, and RS: roles/writing—original draft and writing—review and editing. All authors contributed to the article and approved the submitted version.

## Conflict of Interest

The authors declare that the research was conducted in the absence of any commercial or financial relationships that could be construed as a potential conflict of interest.
