# Supplementary material for: Analysis of COVID-19 Cases' Spatial Dependence in US Counties Reveals Health Inequalities
Source: Front Public Health. 2020 Nov 12;8:579190. doi: 10.3389/fpubh.2020.579190 (PMC7690561; doi:10.3389/fpubh.2020.579190)
Supplement: Supplementary file 1 [file Data_Sheet_1.docx]

Supplementary materials to

**Analysis of COVID-19 cases’ spatial dependence in US counties reveals health inequalities**

T. Saffary^1,†^, OA Adegboye^2,3,†*^, E. Gayawan^4^, F. Elfaki^5^, Md Abdul Kuddus^6^, R. Saffary^7^

# LISA maps based on the adjusted level of significance for multiple comparisons

We used both Bonferroni bounds and false discovery rate (FDR) to adjust the level of significance for the multiple comparisons (correlated tests) across spatial locations [1-3]. In this study, the correction for correlated tests for 3108 contiguous US counties using conservative correction (Bonferroni bounds) erased most of the spatial patterns shown in this study. This is because the number of comparisons equals the number of observations in this study, on the other hand, FDR correction which is less conservative, resulted in more realistic patterns of spatial dependency. See de Castro and Singer (2006) [3] for more details.

**Univariate and bivariate LISA maps based on correction for multiple comparisons (Borerroni bounds and false discovery rate**

| Bonferroni correction | False discovery rate |
| --- | --- |
| Cases per 100,000^†^ |  |
| 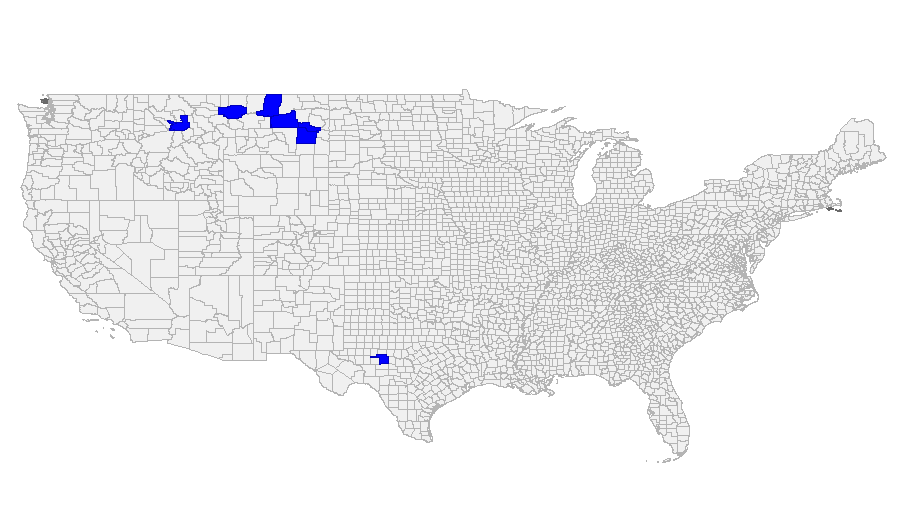 | 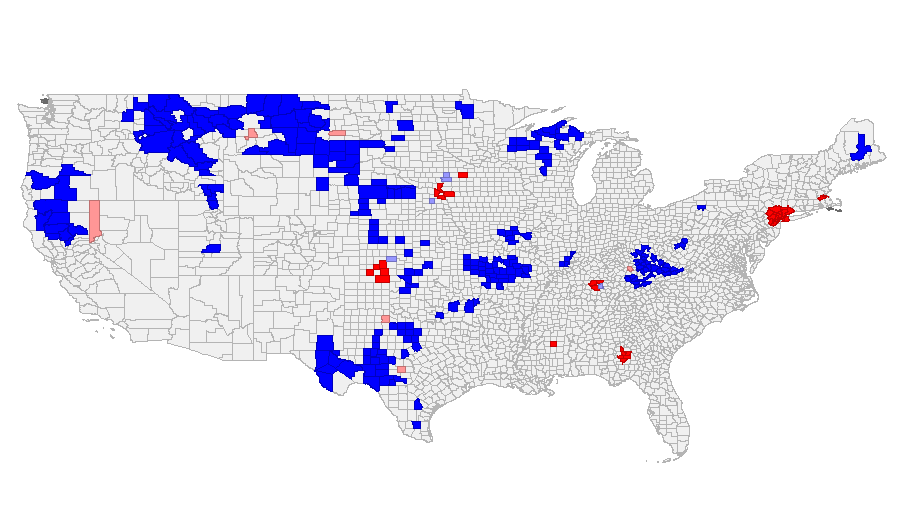 |
| Deaths per 100,000^†^ |  |
| 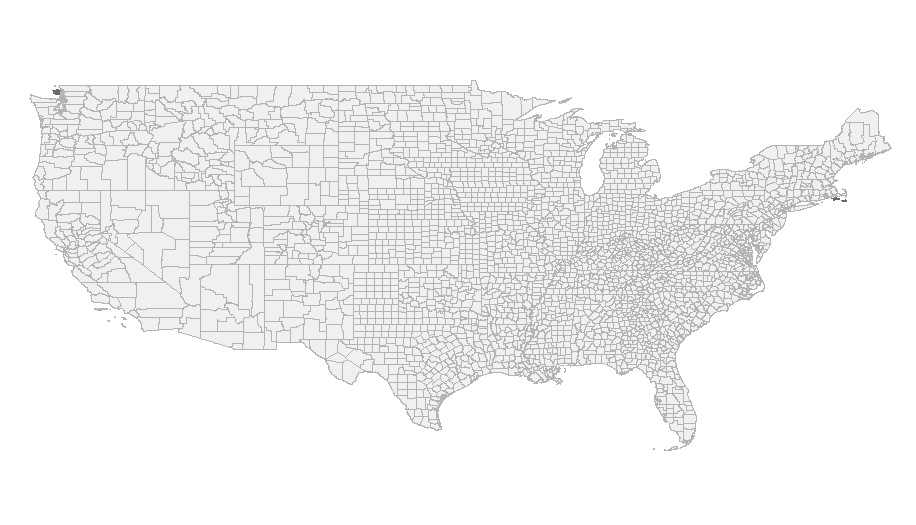 | 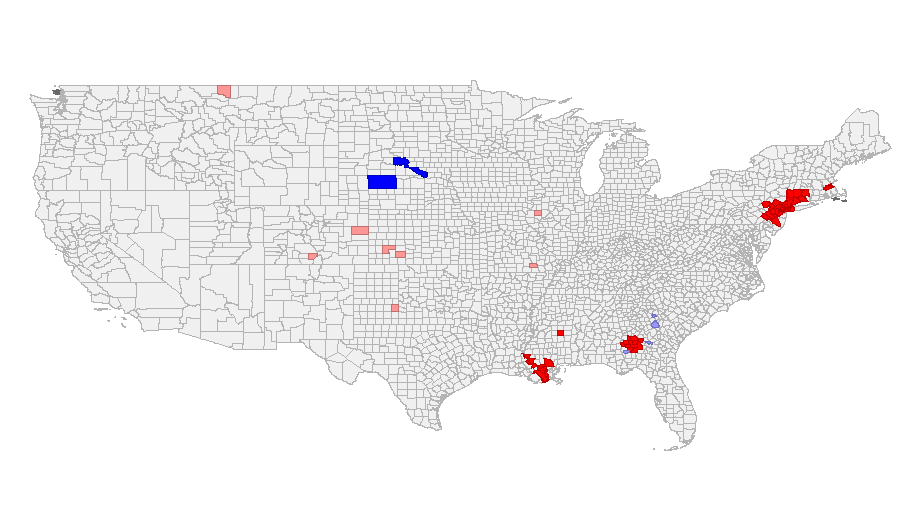 |
| Cases vs ICU^¥^ |  |
| 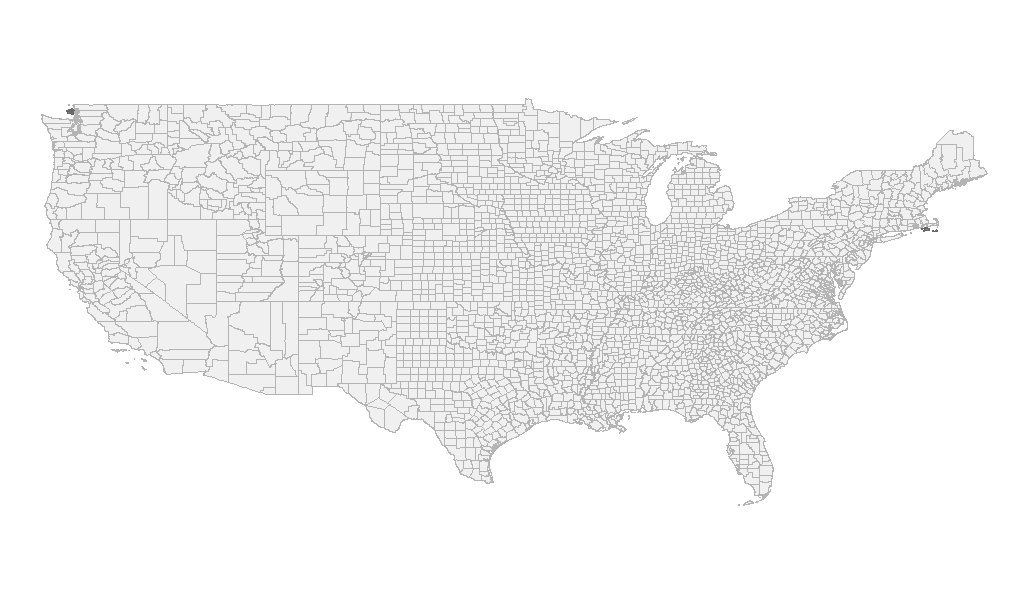 | 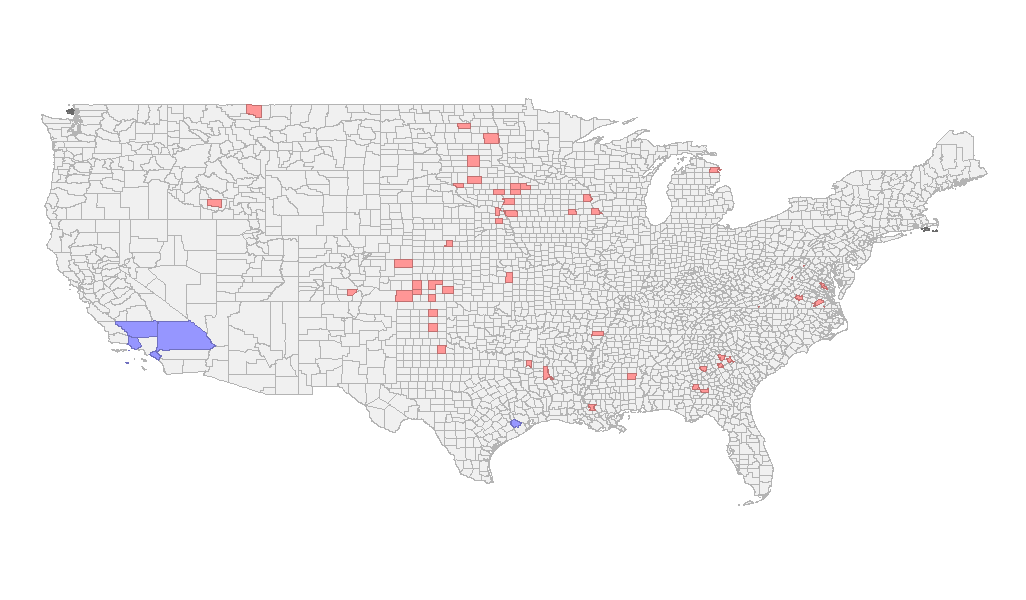 |
| Deaths vs ICU^¥^ |  |
| 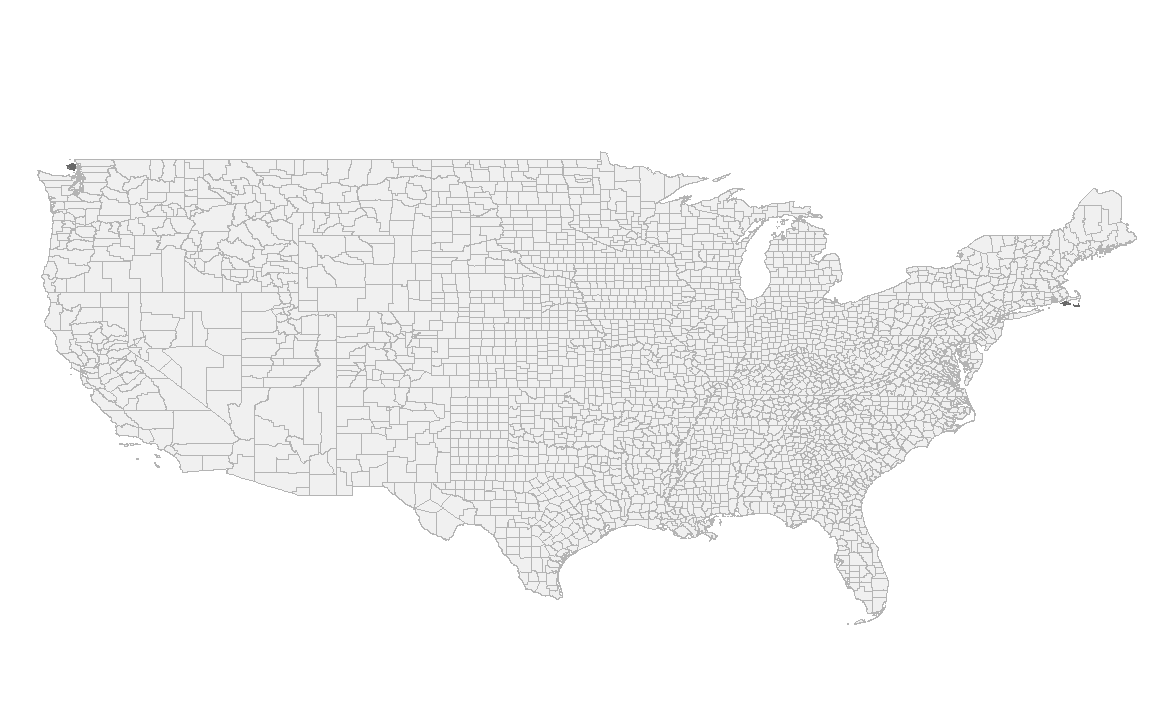 | 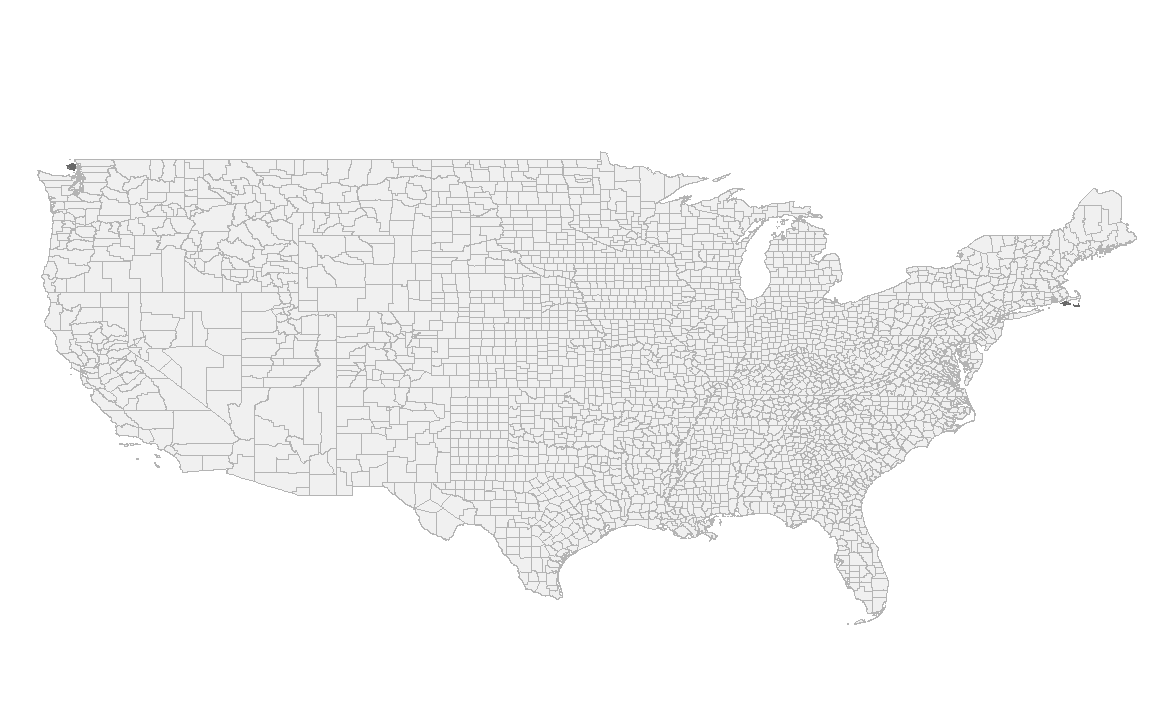 |
| Cases vs Physicians^¥^ |  |
| 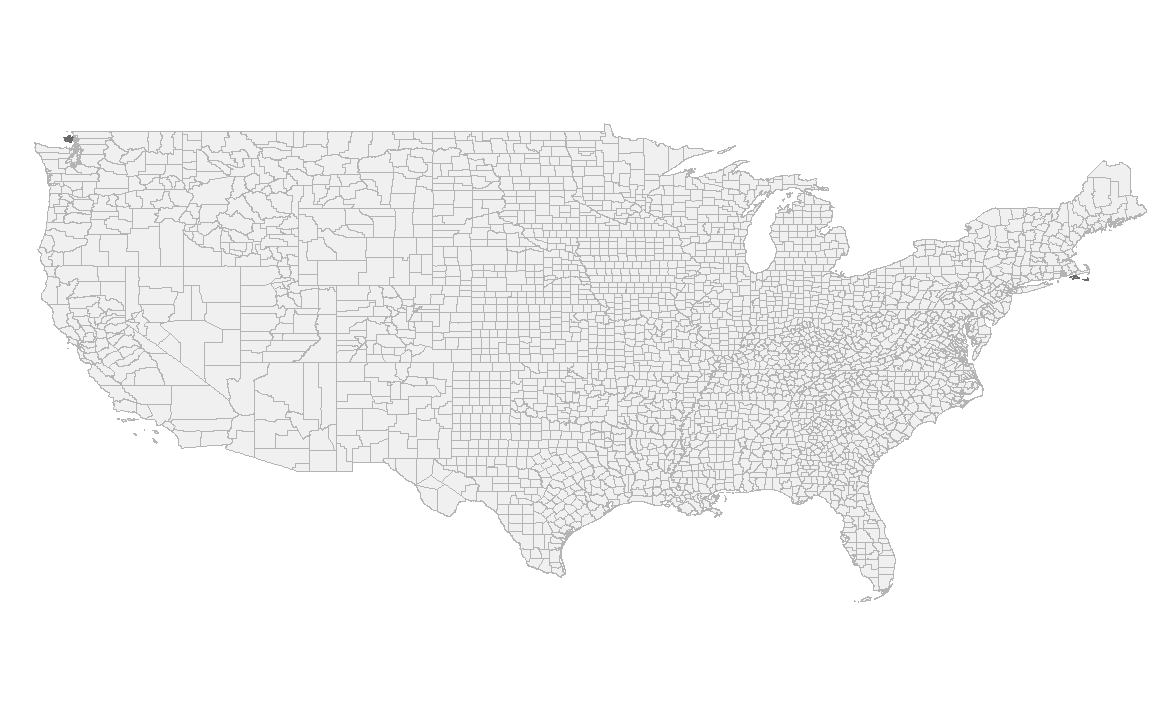 | 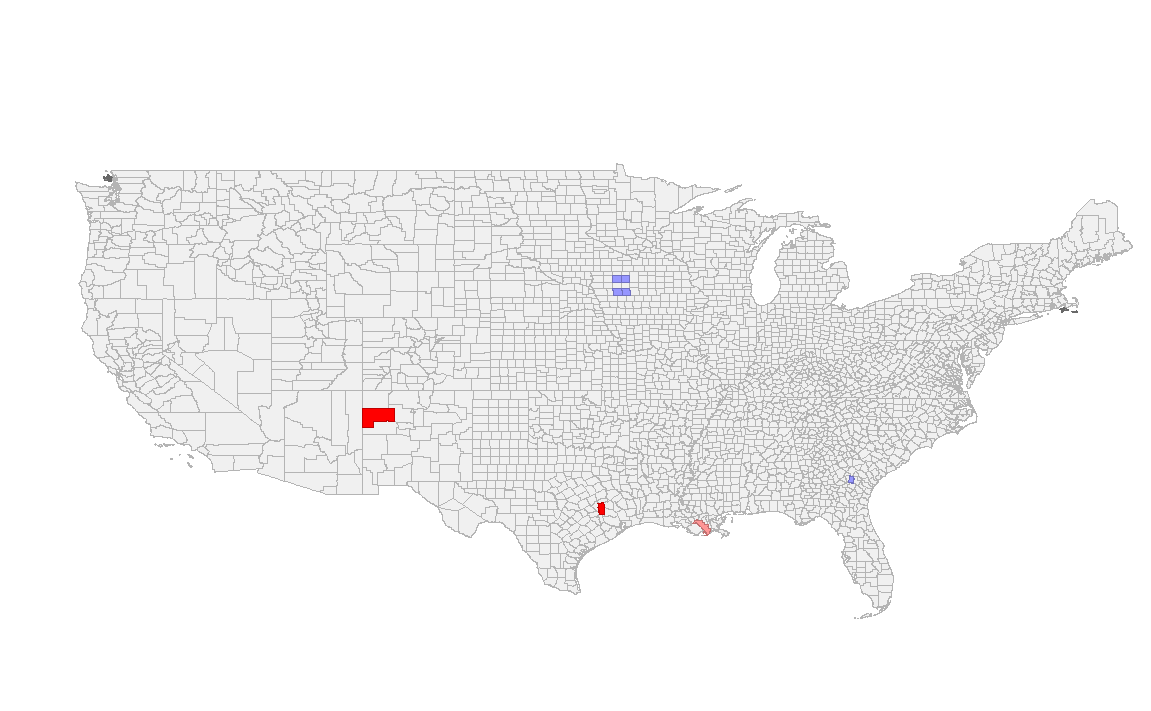 |
| Deaths vs Physicians^¥^ |  |
| 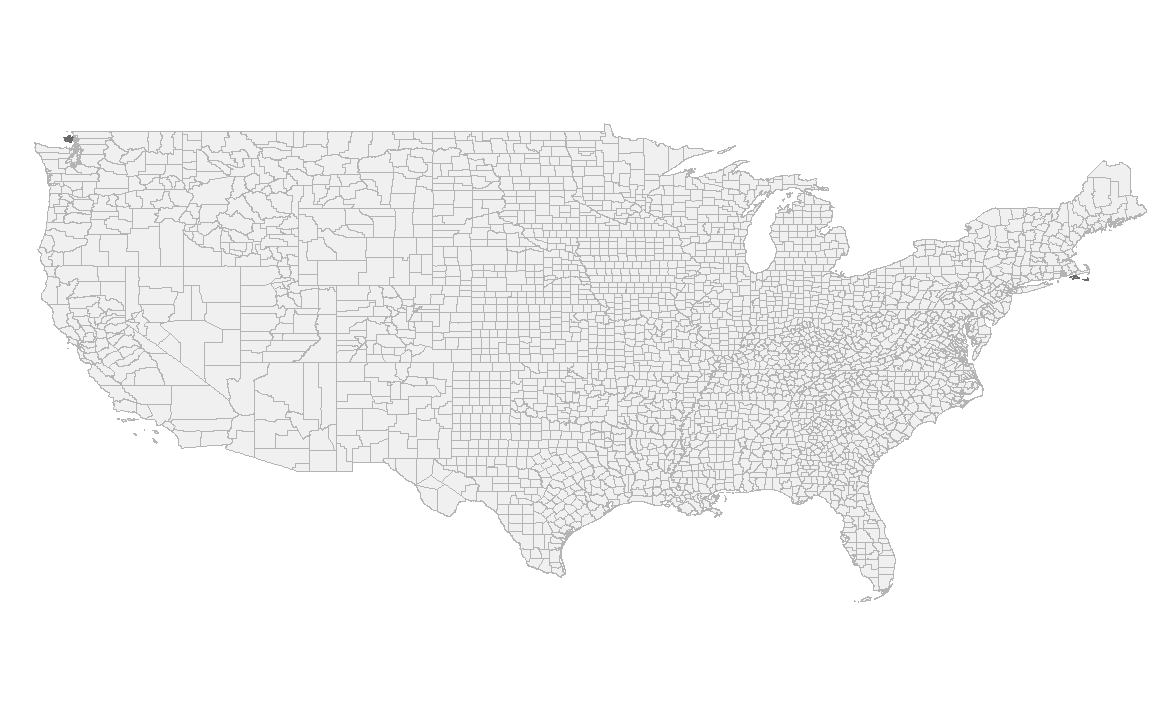 | 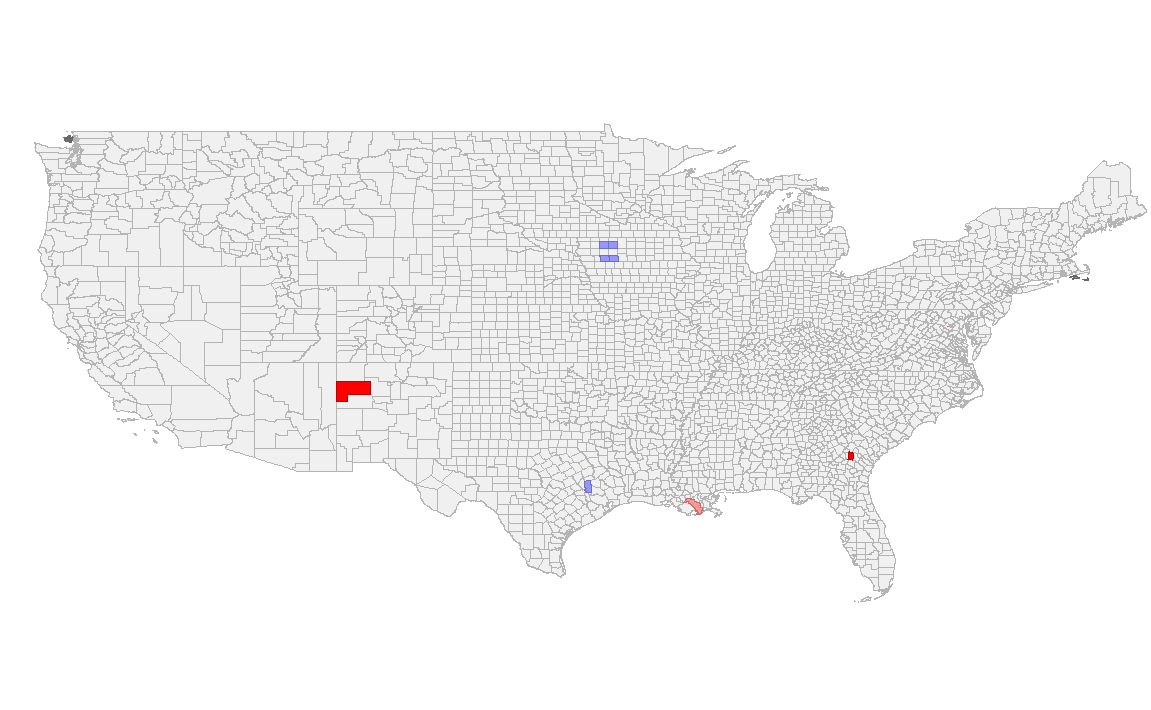 |
| Cases vs Diabetes^¥^ |  |
| 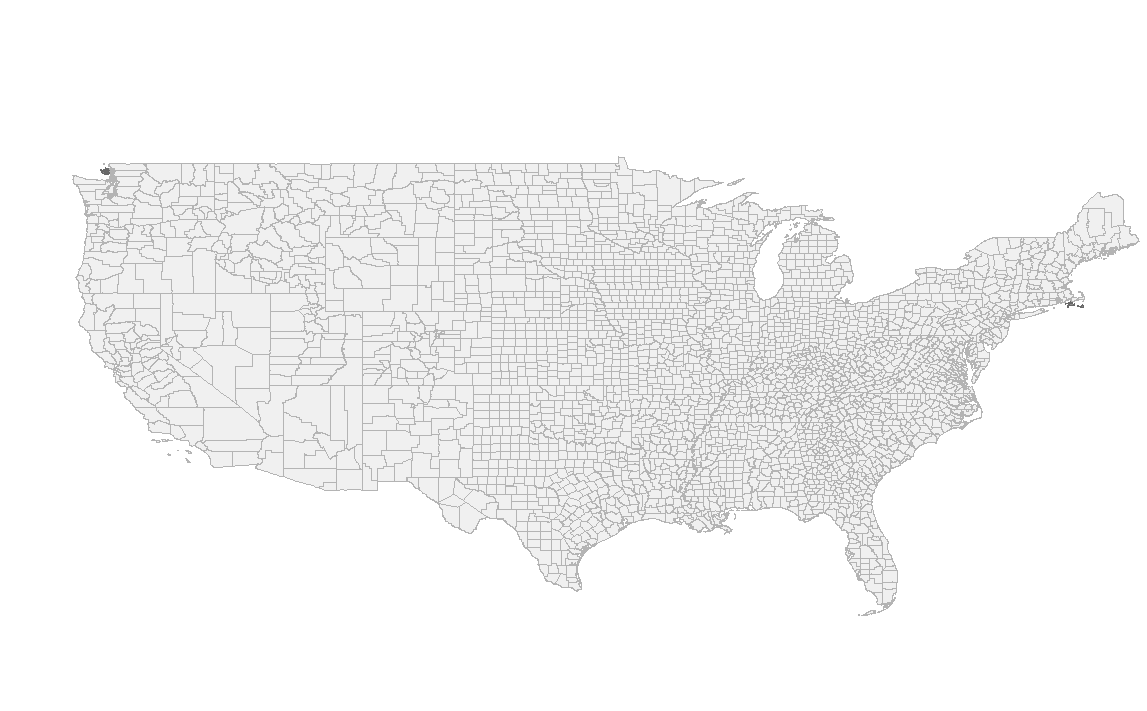 | 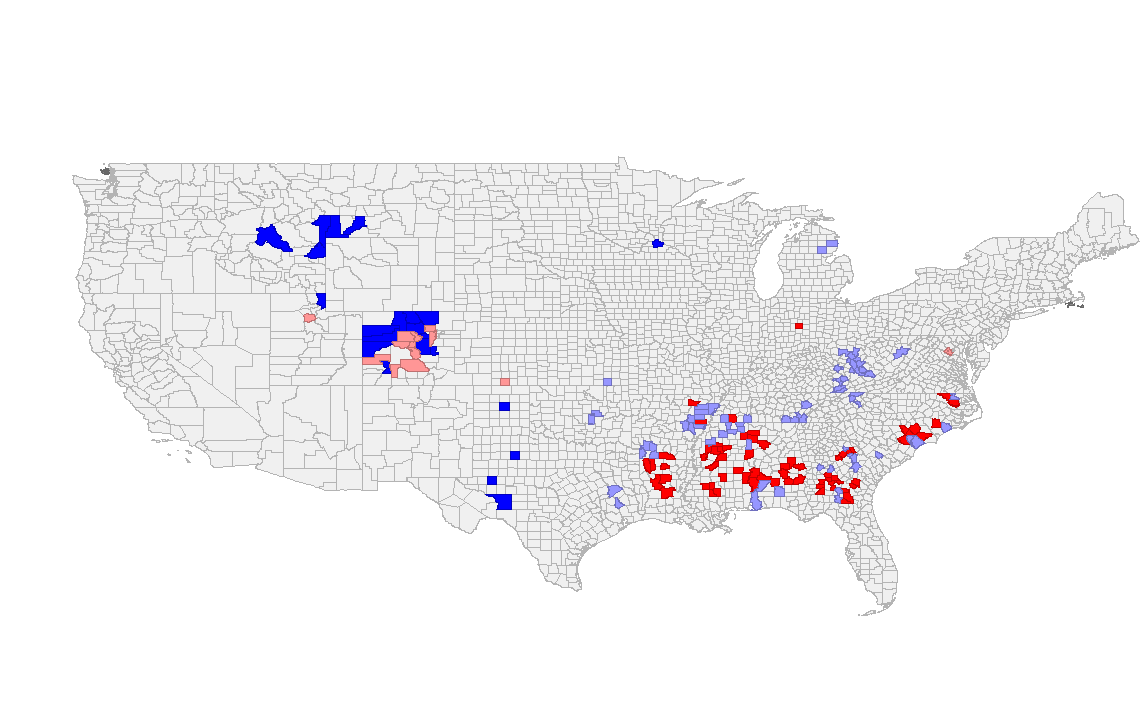 |
| Deaths vs Diabetes^¥^ |  |
| 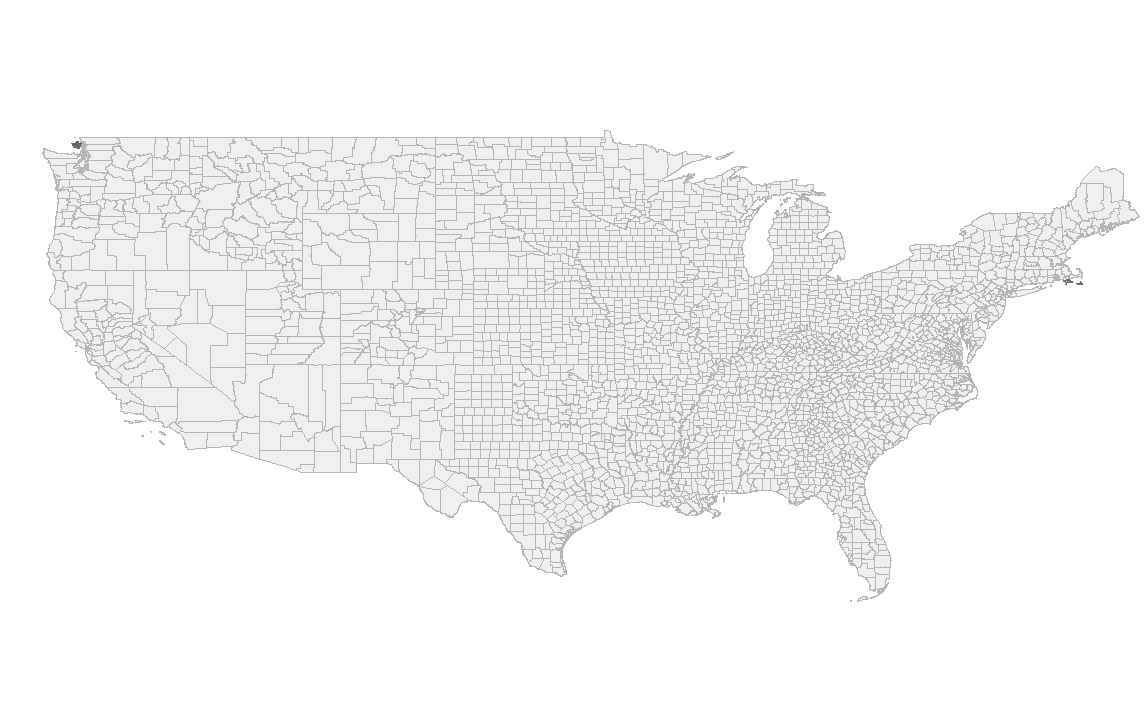 | 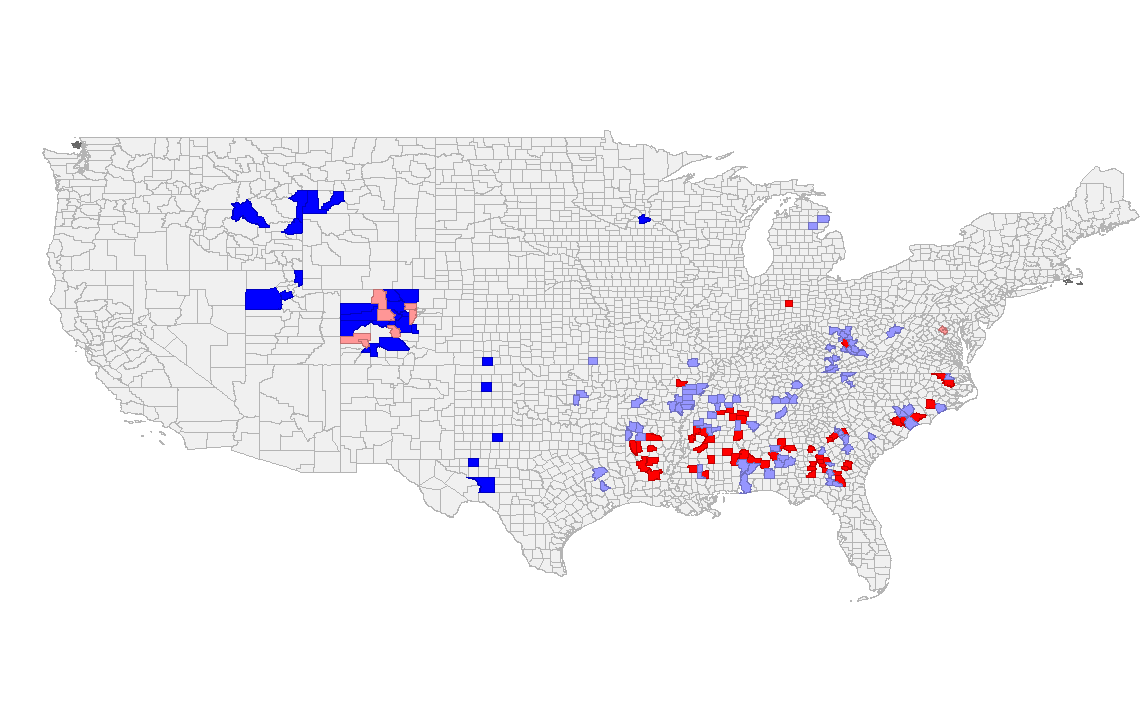 |
| Cases vs African American^¥^ |  |
| 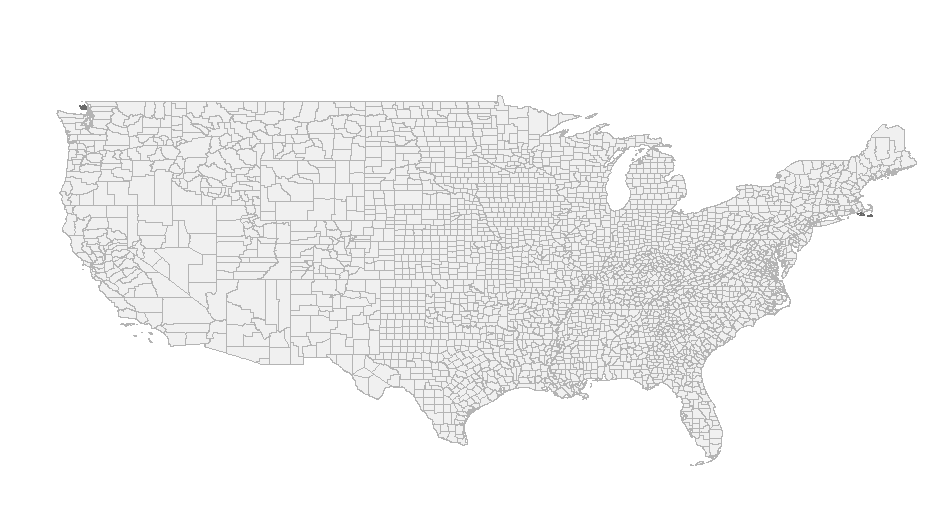 | 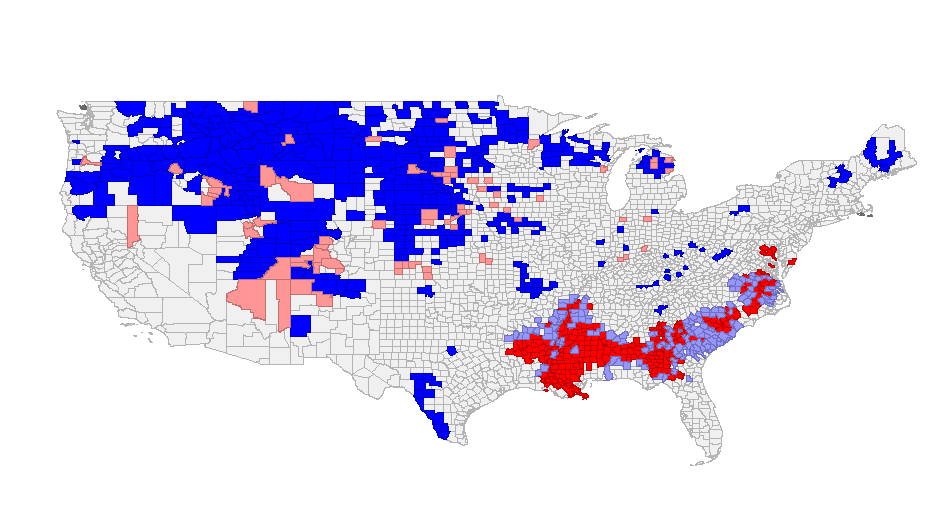 |
| Deaths vs African American^¥^ |  |
| 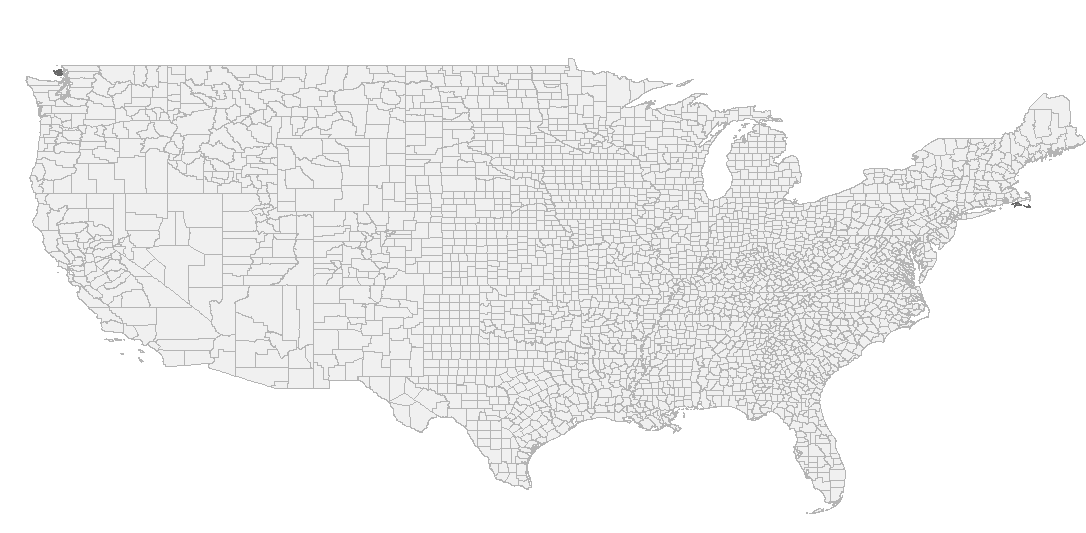 | 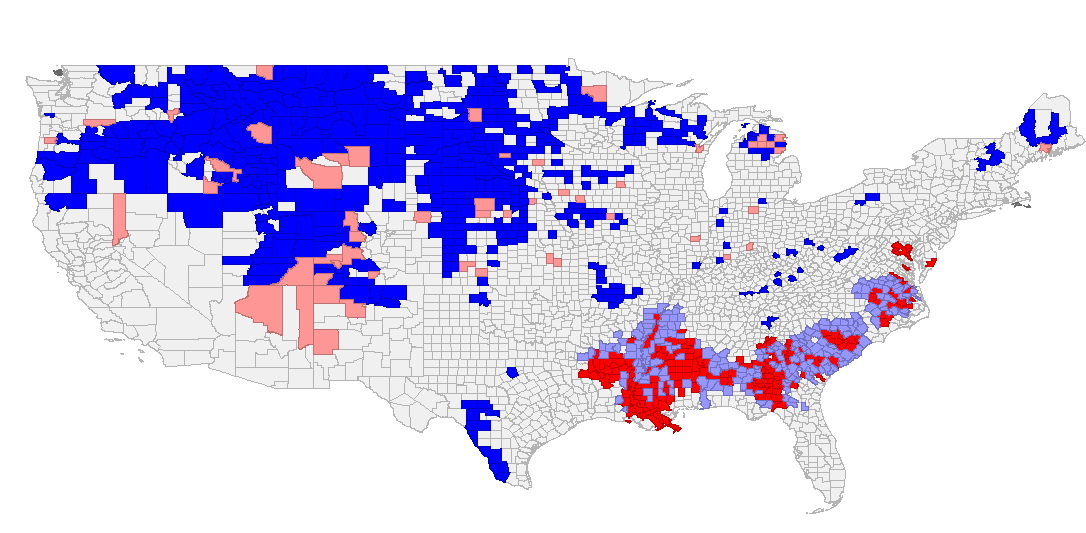 |
| Cases vs Hispanic^¥^ |  |
| 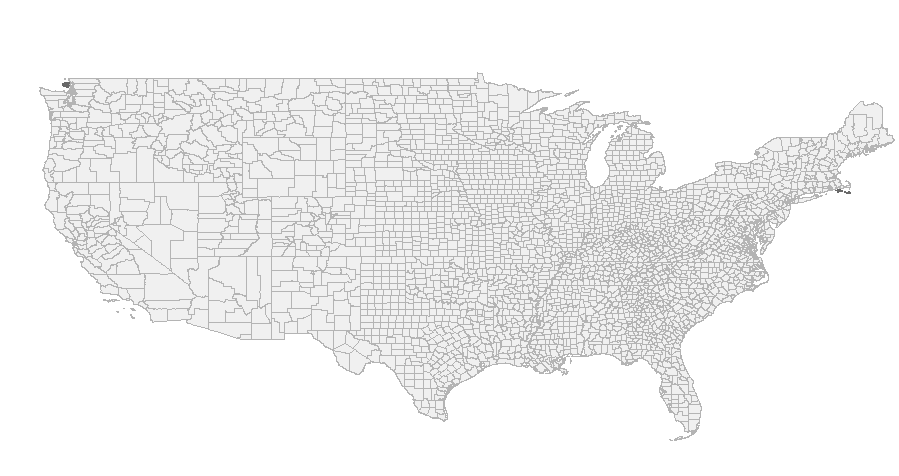 | 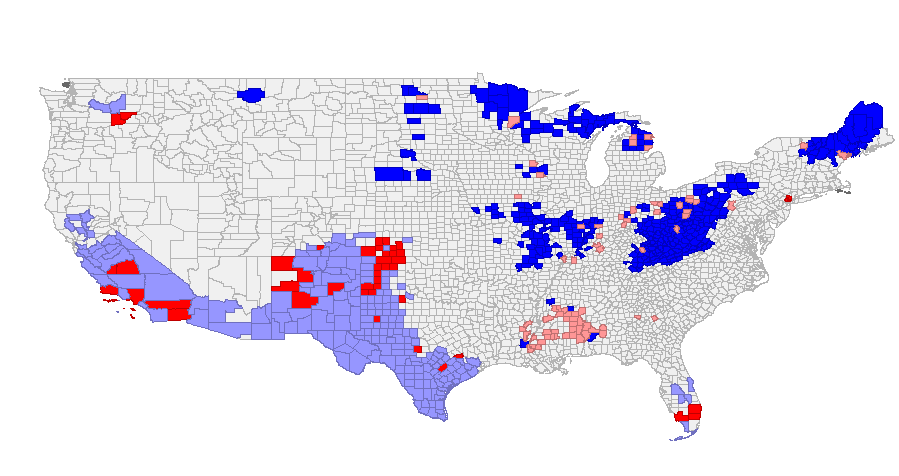 |
| Deaths vs Hispanic^¥^ |  |
| 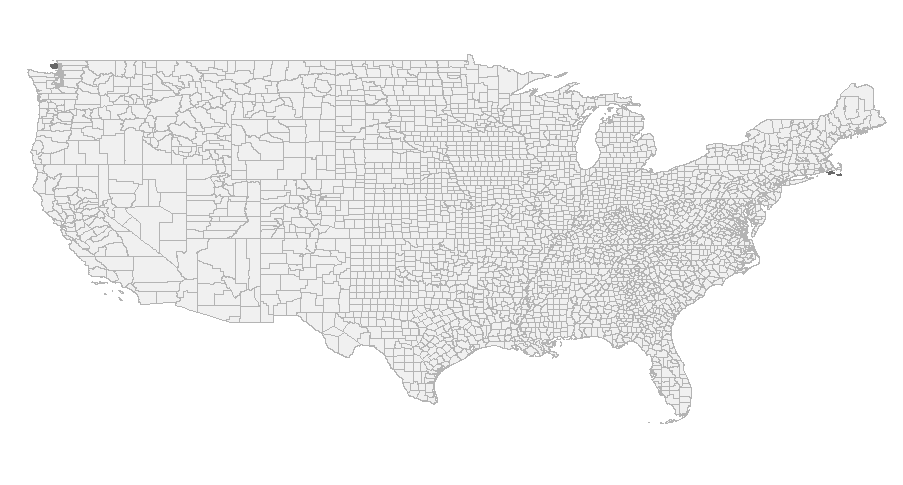 | 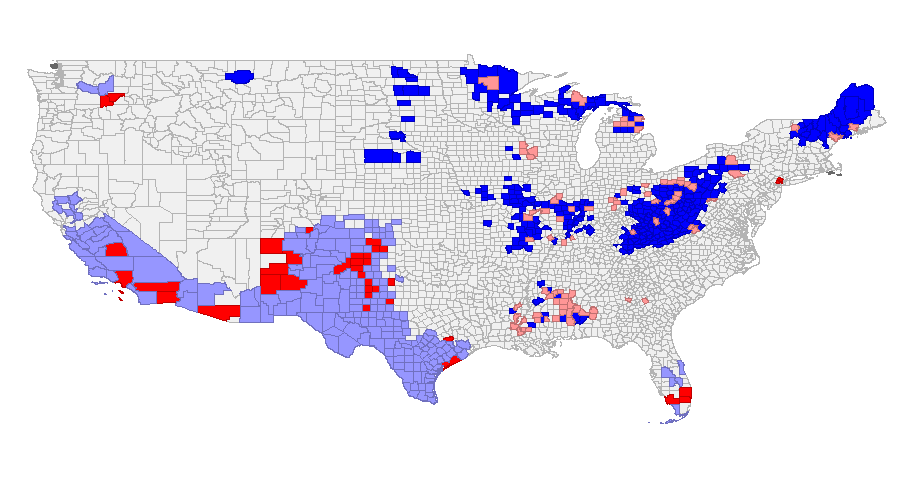 |
| Cases vs White^¥^ |  |
| 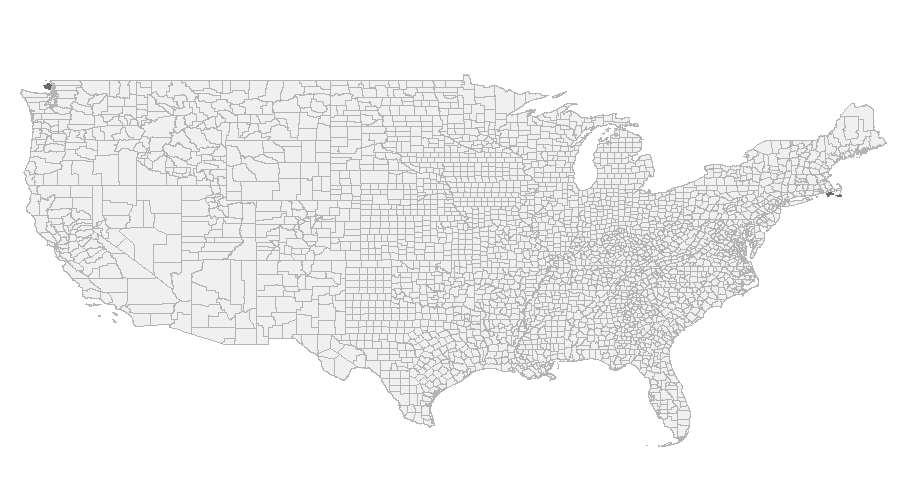 | 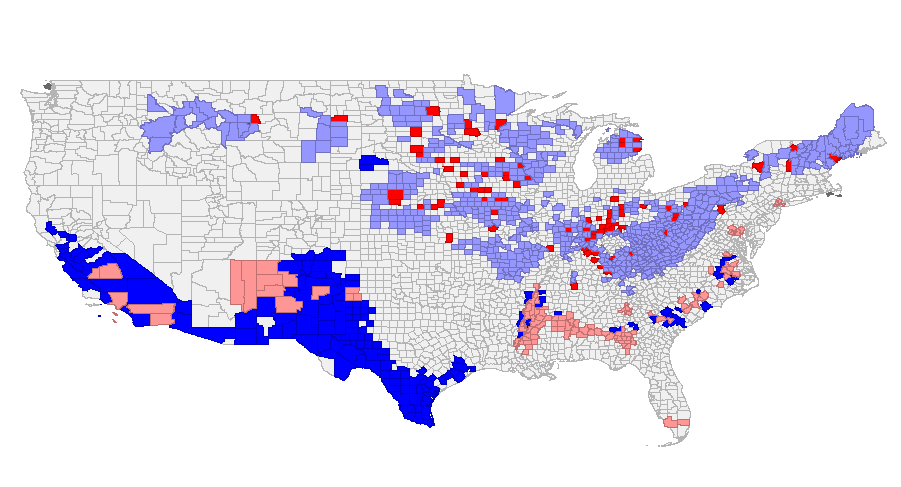 |
| Deaths vs White^¥^ |  |
| 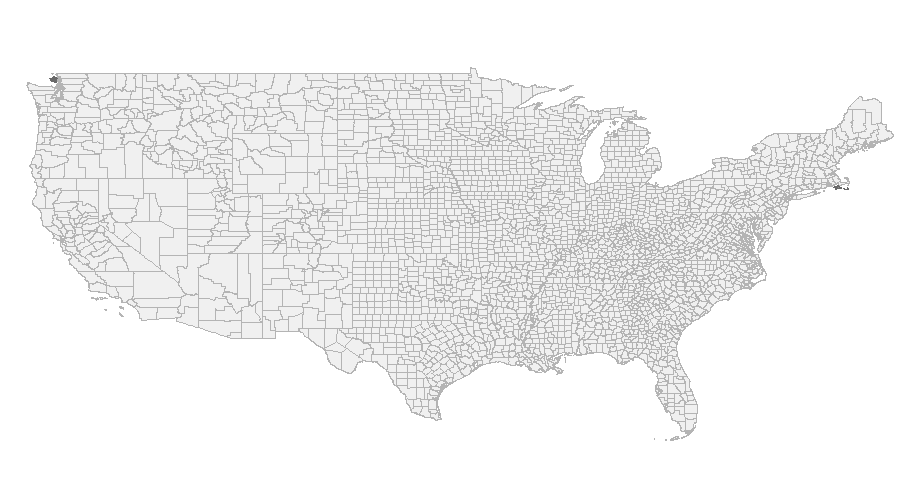 | 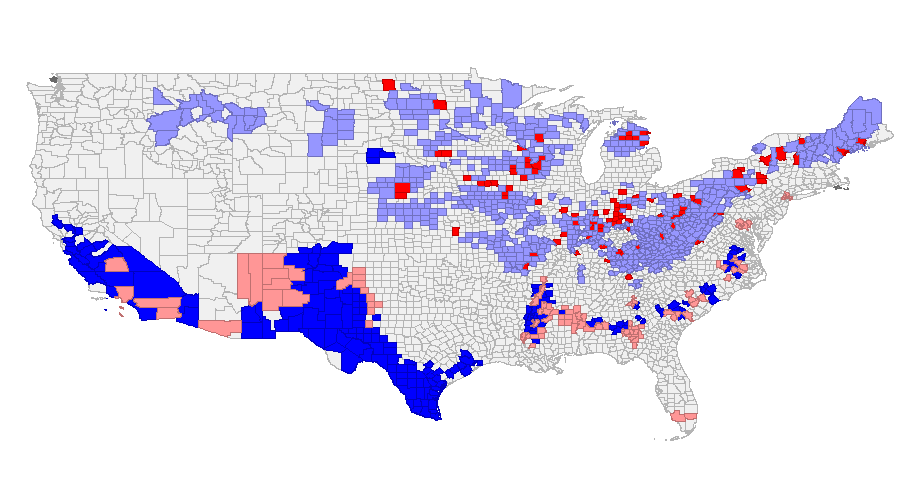 |

^†^Univariate Local *Moran’s I*

^¥^Bivariate Local *Moran’s I*

# References

[1] Anselin L. 1995. Local indicators of spatial association—lisa. Geographical analysis 27:93-115.

[2] Bivand RS, Wong DW. 2018. Comparing implementations of global and local indicators of spatial association. Test 27:716-748.

[3] Caldas de Castro M, Singer BH. 2006. Controlling the false discovery rate: A new application to account for multiple and dependent tests in local statistics of spatial association. Geographical Analysis 38:180-208.
